# 여성노인의 건강관련 삶의 질 수준별 관련요인: 국민건강영양조사(2019년) 자료를 이용한 이차자료분석

**DOI:** 10.4069/kjwhn.2022.06.17

**Published:** 2022-09-07

**Authors:** Miseon Son

**Affiliations:** Design Hospital, Jeonju, Korea; 대자인병원

**Keywords:** Aged, Health status, Quality of life, Women, 노인, 건강상태, 삶의 질, 여성

## Abstract

**Purpose:**

The purpose of this study was to investigate factors related to the levels of health-related quality of life (HRQoL) in elderly women based on Wilson and Cleary’s HRQoL model.

**Methods:**

This study analyzed data from the eighth Korea National Health and Nutrition Examination Survey 2019 on 868 women over the age of 65 years. Based on the HRQoL model, parameters were categorized as personal, environmental, and physiological characteristics; symptom status; functional status; and perception of health status. The data were analyzed by quantile regression.

**Results:**

The overall level of HRQoL was 0.87. Factors related to HRQoL in the 10% quantile were higher education level, higher economic status, economic activity, more walking days, fewer diseases, lower stress, less activity limitation, and higher perceived health status. Factors related to the 25% quantile of HRQoL were more walking days, fewer diseases, less activity limitation, and higher perceived health status. Factors related to the 50% quantile were age, economic activity, more walking days, fewer disease, lower stress, less activity limitation, and higher perceived health status. Factors related to the 75% quantile of HRQoL were smoking, more walking days, fewer diseases, lower stress, less activity limitation, and higher perceived health status.

**Conclusion:**

While differing parameters were identified according to the level of HRQoL of elderly women in Korea, there were five common factors. Interventions that focus on increasing walking, mitigating diseases, stress, and activity limitations, and improving perceived health status can improve HRQoL.

## Introduction

우리나라는 2017년에 만 65세 이상의 고령인구 비율이 14% 이상을 차지하는 고령사회로 진입하였으며, 2025년에는 고령인구의 비율이 20% 이상을 차지하는 초고령사회가 될 것으로 예상되고 있다[1]. 이와 함께 우리나라의 평균 기대수명은 2020년 기준 남성은 80.5세, 여성은 86.5세이나, 건강수명은 남성 65.6세, 여성 67.2세로[2] 노년기에 여성이 남성에 비해 건강하지 않은 상태로 보내는 기간이 길어지고 있어 여성노인의 건강증진에 대한 관심이 필요하다.

노인은 노화나 만성질환의 이환, 직업에서의 은퇴, 사회적 고립 등 다양한 스트레스 상황 및 변화로 인해 건강상태나 삶의 질의 저하를 경험한다[3,4]. 건강관련 삶의 질은 개인이 자신의 신체적, 정신적, 사회적 건강에 대해 인식하는 주관적인 안녕으로[5] 노인의 낮은 건강관련 삶의 질은 질환의 이환이나 사망을 예측할 수 있는 중요한 요인이다[6]. 특히 여성노인의 경우 남성보다 기대수명이 길어 만성질환에 이환되거나 허약한 상태로 보내는 기간이 길어지며[2], 출산 및 육아, 폐경 등으로 인해 상대적으로 낮은 수준의 건강관련 삶의 질을 보일 수 있다[7,8]. 건강관련 삶의 질이 낮을 경우 우울이나 자살 생각이 증가하는 등 여러 문제가 야기될 수 있다[9,10]. 따라서 여성노인의 건강관련 삶의 질을 증진하기 위한 방안 모색이 필요하며, 이를 위해 어떠한 요인들이 건강관련 삶의 질과 관련 있는지 파악할 필요가 있다.

Wilson과 Cleary [5]의 건강관련 삶의 질 모형은 생의학적인 패러다임과 사회과학적인 패러다임을 통합하여 건강관련 삶의 질과 관련된 요인들을 포괄적으로 살펴보았기에 건강을 평가하는 데 중요한 척도로서 활용이 가능하다. 이 모형에서는 건강관련 삶의 질과 관련된 요인으로 생물학적∙생리적 요인, 증상상태, 기능상태 및 전반적인 건강지각을 제시하였으며, 이러한 요인과 건강관련 삶의 질에 개인적 특성과 환경적 특성이 관련 있다고 하였다[5]. 생물학적∙생리적 요인은 객관적으로 관찰되고 확인 가능한 요인으로 동반질환의 수, 만성질환 이환 여부 등이 해당한다[4,11]. 개인이 지각하는 비정상적인 신체적, 정서적, 인지적 상태인 증상상태는 스트레스, 우울, 외로움 등을 포함한다[4,12]. 개인에게 주어진 일을 수행할 수 있는 능력인 기능상태는 활동제한, 기능제한 등을 포함한다[9]. 전반적인 건강지각은 개인이 주관적으로 평가한 전반적인 건강상태로, 주관적인 건강상태가 이에 해당한다[13]. 이외에도 나이, 교육수준 및 소득수준, 경제활동 여부, 흡연 및 음주, 신체활동 등의 개인적 특성[9,12]과 가족의 지지, 사회적 지지, 사회적 관계망 등을 포함한 환경적 특성[11,14]이 노인여성의 건강관련 삶의 질과 관련이 있는 것으로 보고되고 있다. 이처럼 여성노인의 건강관련 삶의 질은 개인의 신체적, 정신적 건강과 더불어 건강생활 신천, 사회‧경제적, 환경적 측면 등 다양한 요인들과 관련이 있으므로 포괄적인 접근이 필요하다.

건강관련 삶의 질 측정을 위한 대표적 도구인 EuroQol 5-Dimension (EQ-5D) 척도는 건강한 상태를 의미하는 최대값으로 치우친 분포를 보인다[15]. 이에 건강관련 삶의 질과 관련요인들의 관계를 파악하기 위해서 선형의 관계를 전재하는 선형회귀모형을 적용하는 데 한계가 있다. 그러나 노인의 건강관련 삶의 질에 대한 선행연구들은 대부분 선형회귀모형을 적용하여 건강관련 삶의 질과 관련요인들과의 관계를 분석하여[3,4,8,9] 정규분포하지 않는 건강관련 삶의 질의 특성을 고려하지 않고 있다. 따라서 정규분포하지 않는 건강관련 삶의 질의 관련요인을 파악하기 위해 분위회귀분석(quantile regression)을 적용하여 건강관련 삶의 질 수준별로 관련 있는 요인을 분석할 필요가 있다[15]. 분위회귀분석은 각 분위 지점에서 다른 분위에 별도의 가중치를 주어 분위별로 회귀모형을 추정하는 분석방법으로, 수준별로 표본을 임의로 분할하여 분석하는 방법과 달리 모든 표본을 이용하여 분석하므로 표본 크기가 작아지거나 임의로 표본을 선택하는 문제가 생기지 않는다[16].

이에 본 연구에서는 전국적인 대표성을 갖는 국민건강영양조사(Korea National Health and Nutrition Examination Survey)의 자료를 사용하여 Wilson과 Cleary [5]의 모형을 토대로 여성노인의 건강관련 삶의 질의 수준별 관련요인을 파악하고자 한다. 이는 여성노인의 건강관련 삶의 질의 증진을 위한 간호중재 방안을 개발하는 데 근거자료로 활용 가능할 것이다.

## Methods

Ethics statement: Obtaining informed consent was exempted by the Institutional Review Board of Wonkwang University (WKIRB-202202-SB-012) because this study was secondary data analysis of existing data and the data were provided in anonymized form.

### 연구 설계

본 연구는 여성노인의 특성에 따른 건강관련 삶의 질의 차이를 확인하고 건강관련 삶의 질과 관련된 요인 파악을 위해 국민건강영양조사의 자료를 이용한 이차자료 분석연구로 기술적 상관성 연구설계이다. 연구의 기술은 STROBE 보고지침(https://www.strobe-statement.org/)에 따라 작성하였다.

### 연구 대상

**Figure 1.**
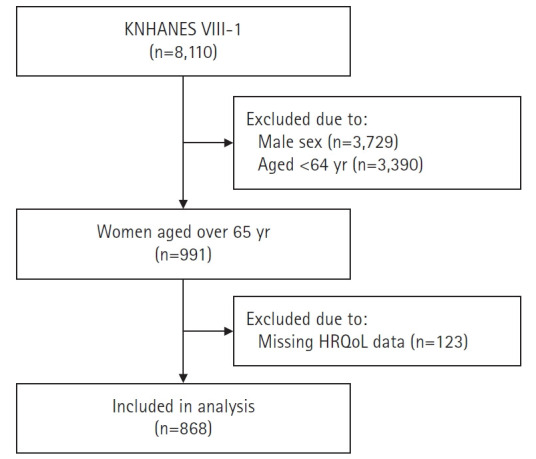
Flow chart of the study. KNHANES: Korea National Health and Nutrition Examination Survey; HRQoL: health-related quality of life.

본 연구는 질병관리청에서 실시한 국민건강영양조사 제8기 1차년도(2019) 조사 자료[17]를 이용하였다. 국민건강영양조사는 조사구 및 가구를 추출단위로 하여 층화집락 표본추출방법을 적용하여 표본을 추출하였다. 국민건강영양조사의 원시자료는 건강설문조사, 검진조사 및 영양조사로 이루어지며, 본 연구에서는 건강설문조사 자료를 이용하였다. 건강설문조사는 이동검진센터에서 면접 방식으로 조사되었으며, 흡연 및 음주 등의 건강행태 영역은 대상자 스스로 자기보고하는 방식으로 조사되었다. 제8기 국민건강영양조사 1차년도(2019년)의 전체 대상자는 8,110명이며, 본 연구에서는 만 65세 이상 여성 991명 중 건강관련 삶의 질 조사에 참여한 868명을 분석 대상자로 선정하였다(Figure 1).

### 자료 수집

본 연구는 국민건강영양조사 제8기 1차년도(2019) 자료 중 연구대상자의 개인식별정보가 포함되지 않은 건강설문조사 자료를 국민건강영양조사 홈페이지(https://knhanes.kdca.go.kr)에서 다운로드하여 사용하였다.

### 연구 도구

본 연구에서는 Wilson과 Cleary [5]의 모형을 기반으로 여성노인의 건강관련 삶의 질과 관련된 요인을 확인하기 위해 주요개념에 따라 개인적 특성, 환경적 특성, 생물학적∙생리적 요인, 증상상태, 기능상태, 전반적인 건강지각으로 구성하였다.

#### 건강관련 삶의 질

건강관련 삶의 질은 한국판 EQ-5D [18] 척도로 측정한 점수를 이용하였다. 본 척도는 운동능력, 자기관리, 일상활동, 통증/불편, 불안/우울, 5개 하위영역으로 구성되어 있다. 각 하위영역은 3점 척도이며, ‘지장이 없다’, ‘다소 지장이 있다’, ‘심하게 지장이 있다’로 구성되어 있다. 각 하위영역에 가중치를 적용하여 단일 점수로 제시하고, 5개 하위영역 모두 ‘지장이 없다’로 응답한 경우 1점으로 계산된다. 점수가 1점에 가까울수록(가능한 범주: –0.17에서 1점) 건강관련 삶의 질 수준이 높음을 의미한다. Lee [19]의 연구에서 본 척도의 신뢰도는 overall percent agreement 값이 79%–97%, kappa 값이 0.32–0.64, intraclass correlation coefficient의 값은 0.61이었다.

#### 개인적 특성

개인적 특성은 나이, 교육수준, 가구소득수준, 경제활동 여부, 흡연 및 음주, 걷기 일수 자료를 이용하였다. 흡연은 ‘현재 담배를 피우십니까’라는 문항에 대한 응답을 이용하였으며, ‘흡연(매일 피움, 가끔 피움)’과 ‘비흡연(피운 적 없음, 과거에는 피웠으나 현재 피우지 않음)’으로 재분류하여 분석하였다. 음주는 ‘최근 1년 동안 술을 얼마나 자주 마십니까?’라는 문항에 대한 응답을 이용하였으며, ‘음주(한 달에 1번 미만, 1번 미만, 1번 정도, 2–4번, 일주일에 2–3번, 4번 이상)’와 ‘비음주(술을 마셔 본 적 없음, 최근 1년간 전혀 마시지 않음)’로 재분류하여 분석하였다. 걷기 일수는 ‘최근 1주일 동안 한 번에 적어도 10분 이상 걸은 날은 며칠입니까?’라는 문항에 대한 응답(0–7일)을 이용하였다.

#### 환경적 특성

환경적 특성에는 가구유형을 포함하였다. ‘세대 유형은 다음 중 무엇에 해당합니까?’에 대한 응답을 ‘1인 가구’와 ‘다인 가구(부부, 부부와 미혼자녀, 편부모와 미혼자녀, 기타)’로 재분류하여 분석하였다.

#### 생리적 요인

생리적 요인에는 동반질환 수를 포함하였다. 30개의 질환(고혈압, 이상지질혈증, 뇌졸중, 심근경색증, 협심증, 골관절염, 류마티스성 관절염, 골다공증, 폐결핵, 천식, 갑상선 질환, 당뇨병, 위암, 간암, 대장암, 유방암, 자궁경부암, 폐암, 갑상선암, 기타 암, 우울증, 아토피피부염, 알레르기비염, 부비동염, 중이염, 신부전, B형 간염, C형 간염, 간경변증, 통풍)에 대한 ‘현재 앓고 있음(현재 유병 여부)’ 문항에 ‘있음’이라고 응답한 질환의 수(0–30개)를 이용하였다.

#### 증상상태

증상상태에는 스트레스를 포함하였다. 스트레스는 ‘평소 일상생활 중에 스트레스를 어느 정도 느끼고 있습니까?’라는 문항에 대해 1점에서 4점까지의 Likert 척도인 ‘대단히 많이 느낌’, ‘많이 느낌’, ‘조금 느낌’, ‘거의 느끼지 않음’으로 응답한 것을 이용하였다.

#### 기능상태

기능상태에는 활동제한 여부를 포함하였다. ‘현재 건강상의 문제나 신체 혹은 정신적 장애로 일상생활 및 사회활동에 제한을 받고 계십니까?’라는 문항에 대해 ‘예’, ‘아니오’로 응답한 것을 이용하였다.

#### 전반적인 건강지각

전반적인 건강지각에는 주관적 건강상태를 포함하였다. ‘평소에 건강은 어떻다고 생각하십니까?’라는 문항을 이용하였으며, ‘좋음(매우 좋음, 좋음)’, ‘보통’, ‘나쁨(나쁨, 매우 나쁨)’으로 재분류하여 분석하였다.

### 자료 분석

자료 분석을 위해 IBM SPSS ver. 26.0 (IBM Corp., Armonk, NY, USA)을 이용하였다. 대상자의 건강관련 삶의 질과 개인적 특성, 환경적 특성, 생리적 요인, 증상상태, 기능상태, 전반적인 건강지각에 해당하는 요인의 빈도 및 백분율, 평균 및 표준편차를 가중치를 적용하여 구하였다. 각 요인에 따른 건강관련 삶의 질의 차이는 복합표본 일반선형모형으로 분석하였다. 복합표본 일반선형모형은 분산분석(analysis of variance, ANOVA)와 t-test 분석이 가능한데, ANOVA의 경우 독립변수 효과가 유의한지에 대해 검정한 것이고, t-test의 경우 독립변수의 각 계수 값이 유의한지에 대해 검정한 것이다. 건강관련 삶의 질 수준별 관련요인을 파악하기 위해 분위회귀분석을 시행하였다. 분위 수 설정을 위해 선행연구[15]를 참고한 결과, 10%, 25%, 50%, 75%, 90% 분위로 나누어 분석을 하였다. 그러나 EQ-5D 척도로 측정한 건강관련 삶의 질의 경우 최대값으로 치우치는 천장효과(ceiling effect)를 보여 본 연구에서는 90% 분위를 제외한 10%, 25%, 50%, 75% 분위로 나누어 상중하, 최하위 집단을 대상으로 분석을 시행하였다.

## Results

### 여성노인의 특성에 따른 건강관련 삶의 질의 차이

본 연구의 대상자는 총 868명이었으며, 여성노인의 건강관련 삶의 질은 0.87±0.17점으로 높지 않은 수준이었다(Table 1).

여성노인의 건강관련 삶의 질은 나이, 교육수준, 가구소득수준, 경제활동 상태, 음주 여부, 걷기 일수, 가구유형, 동반질환 수, 스트레스, 활동제한 여부, 주관적 건강상태에 따라 유의한 차이가 있었다(Table 1). 65–74세 여성이 75세 이상의 여성보다 건강관련 삶의 질이 높았으며(t=4.02, *p*<.001), 교육수준이 초등학교 졸업 이하인 경우가 고등학교 졸업 이상인 경우보다 건강관련 삶의 질이 낮은 것으로 나타났다(t=–7.40, *p*<.001). 가구소득수준이 ‘하’인 경우보다 ‘상’인 경우(t=3.47, *p*=.001)와 ‘중상’인 경우(t=3.10, *p*=.002)에 건강관련 삶의 질이 높았으며, 경제활동에 참여할 경우(t=2.26, *p*=.025), 걷기 일수가 많을수록(t=7.91, *p*<.001) 건강관련 삶의 질이 높은 것으로 나타났다. 1인 가구인 경우(t=–3.43, *p*=.001), 동반질환 수가 많을수록(t=–8.15, *p*<.001) 건강관련 삶의 질이 낮은 것으로 나타났다. 스트레스를 매우 많이 느낀 경우가 거의 느끼지 않는 경우보다 건강관련 삶의 질이 낮았으며(t=–3.38, *p*=.001), 우울감을 느낀 경우(t=–2.48, *p*=.014), 활동제한이 있는 경우(t=–7.54, *p*<.001) 건강관련 삶의 질이 낮은 것으로 나타났다. 주관적 건강상태가 나쁜 경우보다 좋은 경우(t=11.45, *p*<.001), 보통인 경우(t=9.67, *p*<.001)가 건강관련 삶의 질이 높은 것으로 나타났다. 여성노인의 흡연 및 음주 여부에 따른 건강관련 삶의 질의 차이는 유의하지 않았다(Table 1).

### 여성노인의 건강관련 삶의 질 수준별 관련요인

여성노인의 건강관련 삶의 질 수준별 관련요인을 파악하기 위해 분위회귀분석을 실시한 결과 (Table 2), 10% 분위 여성노인의 경우 소득수준이 ‘상’인 경우(B=0.06, *p*=.045), 경제활동에 참여하는 경우(B=0.04, *p*=.010), 걷기 일수가 많을수록(B=0.01, *p*<.001), 주관적 건강상태가 좋은 경우(B=0.22, *p*<.001)와 보통인 경우(B=0.19, *p*<.001) 건강관련 삶의 질이 높은 것으로 나타났으며, 교육수준이 초등학교 졸업 이하인 경우(B=–0.05, *p*=.016), 동반질환 수가 많을수록(B=–0.01, *p*=.021), 스트레스를 매우 많이 느끼는 경우(B=–0.12, *p*=.002) 및 활동제한이 있는 경우(B=–0.19, *p*<.001) 건강관련 삶의 질이 낮은 것으로 나타났다. 25% 분위의 여성노인은 걷기 일수가 많을수록(B=0.01, *p*<.001), 주관적 건강상태가 좋은 경우(B=0.14, *p*<.001)와 보통인 경우(B=0.11, *p*<.001) 건강관련 삶의 질이 높은 것으로 나타났으며, 동반질환 수가 많을수록(B=–0.02, *p*<.001) 및 활동제한이 있는 경우(B=–0.12, *p*<.001) 건강관련 삶의 질이 낮은 것으로 나타났다. 50% 분위의 여성노인은 65–74세인 경우(B=0.03, *p*=.007), 경제활동에 참여하는 경우(B=0.02, *p*=.043), 걷기 일수가 많을수록(B=0.01, *p*=.001), 주관적 건강상태가 좋은 경우(B=0.10, *p*<.001)와 보통인 경우(B=0.08, *p*<.001) 건강관련 삶의 질이 높은 것으로 나타났으며, 동반질환 수가 많을수록(B=–0.01, *p*<.001), 스트레스를 매우 많이 느끼는 경우(B=–0.10, *p*<.001) 및 활동제한이 있는 경우(B=–0.10, *p*<.001) 건강관련 삶의 질이 낮은 것으로 나타났다. 75% 분위의 여성노인은 걷기 일수가 많을수록(B=0.01, *p*<.001), 주관적 건강상태가 좋은 경우(B=0.09, *p*<.001)와 보통인 경우(B=0.09, *p*<.001) 건강관련 삶의 질이 높은 것으로 나타났으며, 흡연하는 경우(B=–0.01, *p*=.008), 동반질환 수가 많을수록(B=–0.01, *p*<.001), 스트레스를 매우 많이 느끼는 경우(B=–0.04, *p*<.001) 및 활동제한이 있는 경우(B=–0.09, *p*<.001) 건강관련 삶의 질이 낮은 것으로 나타났다.

## Discussion

본 연구 결과 건강관련 삶의 질과 관련된 요인 중 수준별 공통요인은 개인적 특성 중 걷기 일수, 생물학적∙생리적 요인인 동반질환 수, 증상상태인 스트레스, 기능상태인 활동제한 여부, 전반적인 건강지각인 주관적 건강상태로 나타났다. 이는 노인의 걷는 시간이 길수록[12], 동반질환이 없는 경우[14], 스트레스가 적을수록[9], 활동제한이 없는 경우[13], 주관적 건강상태가 좋을수록[4] 건강관련 삶의 질이 증가한다고 보고한 연구들과 유사한 결과이다. 건강관련 삶의 질 수준별 차이를 보인 개인적 특성을 살펴보면, 10% 분위인 여성노인의 경우 개인적 특성 중 교육수준 및 가구소득수준, 경제활동이 건강관련 삶의 질과 관련이 있는 것으로 확인되었다. 이는 노인의 교육수준 및 소득수준이 높을수록 [12], 경제활동에 참여하는 경우[12] 건강관련 삶의 질이 증가한다고 보고한 연구들과 유사한 결과이다. 50% 분위인 경우 여성노인의 건강관련 삶의 질은 나이, 경제활동 여부와 관계가 있는 것으로 확인되었다. 이는 노인의 나이가 적을수록[4], 경제활동에 참여하는 경우[12] 건강관련 삶의 질이 증가하는 것으로 보고한 연구들과 유사한 결과이다. 75% 분위인 경우 여성노인의 흡연 여부가 건강관련 삶의 질과 관련된 요인으로 나타났다. 이러한 결과는 노인이 흡연하는 경우 건강관련 삶의 질이 낮다고 보고한 연구[9]와 맥락을 같이한다.

본 연구 결과 여성노인의 개인적 특성 중 걷기 일수는 건강관련 삶의 질의 모든 수준에서 관련요인으로 나타났다. 여성은 나이가 들어감에 따라 만성질환에 쉽게 이환되고, 활동에 제한이 발생하면서 전반적으로 건강상태가 저하되는데 신체활동은 이러한 변화에 영향을 미칠 수 있다[13,20,21]. 그러나 여성노인의 경우 체력 및 근골격계 건강 저하, 상해 위험 증가 등으로 인해 고강도의 운동에는 어려움이 있으므로 걷기운동처럼 비교적 강도가 낮고 경제적 부담 없이 혼자서도 쉽게 할 수 있는 운동이 건강증진과 삶의 질 향상에 도움이 될 수 있다[12,22]. 규칙적인 걷기운동은 신체적 건강의 증진뿐만 아니라 기분 전환, 스트레스나 우울감 감소와 같이 정신적 건강에도 긍정적인 영향을 미칠 수 있다[14,23]. 따라서 여성노인의 건강관련 삶의 질을 향상하기 위해 걷기운동과 같은 신체활동이 중요한 역할을 할 수 있으므로 여성노인에게 이러한 활동에 참여하는 것이 중요한 이유를 교육하고, 여성노인의 신체활동을 위한 지역사회의 노력이 필요하다.

본 연구 결과, 여성노인의 건강관련 삶의 질의 모든 수준에서 생물학적∙생리적 요인인 동반질환 수도 관련요인으로 확인되었다. 노인에게 발생한 질환의 수가 증가할수록 질환 간, 혹은 약물들이 영향을 미쳐 건강상태가 악화될 수 있다[24]. 또한 여성노인에게 발생한 질환은 일상생활 및 활동의 제한, 질환의 관리 및 치료를 위한 경제적 부담, 가족에 대한 의존도 및 심리적 부담감 증가로 인해 건강관련 삶의 질뿐만 아니라 전반적인 삶의 만족에도 부정적 영향을 미칠 수 있다[4,14,25]. 따라서 여성노인에게 발생 가능한 질환을 조기에 발견하고 예방함으로써 복합적으로 질환에 이환되지 않도록 관리하고, 이미 이환된 질환의 경우 적절히 관리하여 전반적인 건강상태나 건강관련 삶의 질 저하를 야기하지 않도록 중재를 제공할 필요가 있다.

여성노인의 스트레스 수준도 건강관련 삶의 질과 관련된 요인으로 나타났다. 여성노인의 경우 건강문제, 가족과의 관계, 경제적 어려움, 은퇴 및 상실감 등으로 인한 심리사회적 문제 등과 관련하여 다양한 스트레스 상황을 경험하게 된다[26]. 스트레스는 우울 등의 정신적인 측면뿐만 아니라 심리적 위축이나 의욕상실 등으로 신체적인 건강에도 부정적 영향을 미침으로써 건강관련 삶의 질 저하를 야기할 수 있다[12,27,28]. 그러나 스트레스 완화를 위해 신체활동을 하거나, 스트레스 상황에 적절히 대처하기 위한 충분한 사회적 지지가 있을 경우 스트레스는 감소할 수 있다[23,27,28]. 따라서 여성노인에게 스트레스를 유발하는 요인을 파악하고, 이러한 요인을 적절히 관리하고 스트레스 상황에 대처하기 위한 여성노인의 능력을 강화하고 활용 가능한 사회적 자원을 연계하는 방안의 모색이 필요하다.

본 연구 결과 모든 건강관련 삶의 질 수준에서 여성노인에게 활동제한이 있는 경우 건강관련 삶의 질이 저하되는 것으로 확인되었다. 나이가 들어감에 따라 여성은 점차 체력이 저하되고 만성질환에 이환되면서 활동하는 데 제한이 발생한다[29]. 활동제한이 있을 경우 일상생활이나 건강관리에 있어 자립적인 생활이 어려워져 가족이나 기타 돌봄제공자에 의지하게 되고, 사회활동 및 여가생활 등이 어려워져 심리사회적 건강도 저하됨에 따라 건강관련 삶의 질 수준이 감소할 수 있다[9,13,20]. 따라서 여성노인의 건강관련 삶의 질을 향상하기 위해 활동제한 정도를 확인하고 활동 및 기능 상태를 향상할 수 있는 중재와 함께 활동이 제한된 상태에서 적응하며 살 수 있도록 사회적 자원을 연계해주는 방안이 필요하다.

이처럼 여성노인은 질환으로 인한 건강상태 저하, 신체적, 심리사회적 문제로 인한 스트레스, 활동 및 기능의 제한 등의 여러 부정적인 변화들을 경험하게 되며 이로 인해 주관적으로 인식하는 자신의 건강상태가 낮아질 수 있다[21,22,30]. 본 연구에서도 여성노인의 건강관련 삶의 질 수준에 관계 없이 주관적 건강상태가 좋을수록 건강관련 삶의 질이 높아지는 것으로 나타났다. 주관적 건강상태에 따라 건강관리를 위한 행위가 결정될 수 있으므로[30] 여성노인이 자신의 건강상태가 나쁘다고 인식할 경우 건강과 관련한 주관적 안녕이 저해될 수 있다[13]. 노화나 질환 등으로 건강상태가 나쁘더라도 건강관리를 위해 적절한 건강 행위를 한다면 주관적으로 인식하는 건강상태는 향상될 수 있다[30]. 이에 여성노인의 주관적 건강상태를 사정하여 낮을 경우에는 우선적으로 직면하고 있는 건강문제를 해결하기 위한 중재를 제공하고, 이와 더불어 신체적, 정신적, 사회적 건강을 증진하고 질환 예방 및 건강 관리를 위한 방안을 마련함으로써 주관적 건강상태와 함께 건강관련 삶의 질도 향상시킬 수 있으리라 생각한다.

본 연구 결과, 건강관련 삶의 질 수준이 중간 정도에 해당하는 50% 분위 여성노인에서 나이가 적을수록 건강관련 삶의 질이 높은 것으로 나타났다. 여성노인이 나이가 들수록 신체적 노화 및 질환의 이환, 건강하지 않은 생활습관, 사회적 위축 등으로 인해 건강관련 삶의 질이 저하될 수 있다[3,4,12]. 그러나 본 연구에서 하위, 상위 집단 여성노인에서는 나이가 건강관련 삶의 질과 관련이 없는 것으로 나타났다. 이러한 결과는 하위 집단의 경우 사회‧경제적 위치와 관련이 있으며, 상위 집단의 경우 비흡연 등의 건강생활 신천과 관련이 있어 나이가 미치는 영향력이 상대적으로 감소한 것으로 생각한다[9,12]. 이에 후기노인 여성, 특히 건강관련 삶의 질 수준이 중간 정도인 여성노인의 건강관련 삶의 질이 저하되지 않도록 관심을 가지고 중재를 제공할 필요가 있다.

본 연구에서 교육수준과 소득수준은 건강관련 삶의 질 수준이 10% 분위인 여성노인에서 건강관련 삶의 질과 관계가 있는 요인으로 확인되었다. 높은 수준의 교육수준이나 가구소득은 건강 문제와 관련하여 보다 나은 대처 능력을 제공하여 건강관련 삶의 질 향상에 기여하게 된다[12]. 그러나 사회‧경제적 위치가 낮으면 건강생활 신천이나 건강관리를 위한 자원에 접근하기가 어려워 건강관련 삶의 질이 저하될 수 있다[13]. 이에 사회‧경제적으로 취약한 여성노인의 경우 건강관련 삶의 질이 더욱 저하될 수 있으므로 집중적인 관리가 필요하다.

여성노인의 경제활동 여부는 건강관련 삶의 질 수준이 최하위인 집단과 중간 집단에서 관련요인으로 나타났다. 경제활동에 참여하는 것은 경제적 측면뿐 아니라 사회활동에 참여한다는 측면에서도 자신의 건강관련 삶의 질이 중간 수준 이하라고 인식하는 여성노인들에게 영향을 줄 수 있다. 사회구성원으로 경제활동에 참여함으로써 자신의 역할을 수행하게 되고 다른 사람들과 상호작용하면서 신체적, 정신적, 사회적 건강에 대한 주관적 인식이 좋아질 수 있다[12]. 여성노인은 남성에 비해 사회‧경제적 기능 측면에서 삶의 질이 낮으며 사회심리적으로 고립되고 외로움을 더욱 느낄 수 있으므로 관심을 가질 필요가 있다[4,8]. 이에 여성노인의 경제활동 참여율을 높이기 위한 정책적 지원을 통해 건강관련 삶의 질을 향상시킬 수 있으리라 생각한다.

흡연은 건강 문제뿐만 아니라 신체활동 등의 건강증진활동과 관련된 주요 요인 중 하나이다[19]. 특히 본 연구결과 건강관련 삶의 질이 높은 수준인 75% 분위 여성노인에서 흡연 여부가 건강관련 삶의 질과 관련된 것으로 나타났다. 이는 건강관련 삶의 질이 높은 여성노인의 경우 비흡연이나 금연과 같은 건강생활을 실천하면서 자신의 건강에 대해 긍정적으로 인식하고 적극적으로 건강관리를 하여 건강관련 삶의 질이 더욱 향상될 수 있음을 의미하는 것으로 볼 수 있다[26]. 이에 높은 수준의 건강관련 삶의 질을 보이는 여성노인의 경우 높은 수준을 유지하는 데 비흡연과 금연 등의 건강생활 신천이 도움이 될 수 있으므로 여성노인의 건강생활 신천을 위한 지원 사업을 마련할 필요가 있다.

본 연구는 국민건강영양조사의 이차자료를 이용함에 따라 여성노인의 건강관련 삶의 질과 관련된 요인들을 측정한 도구가 단일문항으로 구성되어 Wilson과 Cleary [5]의 모형에서 제시한 관련요인들의 특성을 반영하는 데 한계가 있으며, 환경적 특성 중 사회적 지원의 직접적인 관계를 확인하지 못했다는 제한점이 있다. 추후 연구에서는 여성노인의 건강관련 삶의 질의 관련요인의 특성을 명확히 반영한 측정도구를 활용하고, 사회적 지원과의 관련성을 파악하기를 제언한다. 이러한 제한점에도 불구하고 본 연구는 여성노인의 건강관련 삶의 질을 증진시키기 위해 고려해야 할 관련요인을 건강관련 삶의 질을 수준별로 나누어 확인하였으며, 포괄적인 접근을 위해 Wilson과 Cleary [5]의 모형을 적용하였다는 의의가 있다

여성노인은 남성노인에 비해 기대수명은 길고 건강수명은 짧아 이들의 건강관련 삶의 질에 관심을 가질 필요가 있다. 노인기 여성의 건강관련 삶의 질은 단순히 신체적 건강과 관련한 요인뿐만 아니라 다양한 요인들에 의해 복합적인 영향을 받으므로 포괄적으로 접근해야 한다. 여성노인의 건강관련 삶의 질 수준별 관련된 요인은 공통적인 부분도 있으나 수준별로 상이한 관련요인도 있으므로 건강관련 삶의 질의 수준에 따라 차별화된 접근이 필요하다. 여성노인 전반에 대해서는 이환된 질환이 많거나, 스트레스나 활동제한 등의 취약성이 있는 경우 직면하고 있는 신체적, 정신적, 사회적 건강문제를 해결하기 위한 중재를 제공할 필요가 있다. 특히 사회‧경제적으로 취약한 여성노인의 경우 낮은 수준의 건강관련 삶의 질을 보일 수 있으며 취약한 사회‧경제적 상황이 유지될 경우 건강관련 삶의 질이 더욱 저하될 수 있기에 집중적인 관리가 필요하다. 또한 건강관련 삶의 질 수준이 중간 정도인 여성노인 중 나이가 많을수록 건강관련 삶의 질이 낮아질 수 있으므로 후기노인 여성에 대한 집중관리도 필요하리라 생각한다. 이러한 집중관리 대상을 포함한 모든 여성노인에게 걷기운동 및 경제활동에 참여할 수 있도록 지원하며, 질환 및 스트레스 관리, 주관적 건강상태 향상을 위한 관리 방안을 제공함으로써 다양한 수준의 건강관련 삶의 질을 보이는 여성노인들에게 전반적인 증진 효과를 기대할 수 있을 것이다. 높은 수준의 건강관련 삶의 질을 보이는 여성노인의 경우 이와 더불어 금연과 같은 건강생활 신천을 수행하도록 지원하여 높은 수준의 건강관련 삶의 질을 유지하는 차별화된 접근이 필요하리라 생각한다.

## Figures and Tables

**Table 1.** Health-related quality of life according to participant characteristics (N=868; weighted N=3,902,210)

Variable	Categories	n (%) or mean±SD	Weighted n (weighted %)	EQ-5D index		
				Mean±SD	t (*p*)	F (*p*)
		0.87±0.17				
*Characteristics of the individual*						
Age (year)	65–74	522 (60.1)	2,148,817 (55.1)	0.89±0.15	4.02 (<.001)	
	≥75	346 (39.9)	1,753,393 (44.9)	0.83±0.18		
Education	≤Elementary	587 (67.8)	2,613,722 (67.1)	0.85±0.18	–7.40 (<.001)	28.00 (<.001)
	Middle school	124 (14.3)	548,233 (14.1)	0.89±0.16	–1.54 (.125)	
	≥High school	155 (17.9)	735,857 (18.8)	0.92±0.11		
Economic status	Very high	64 (7.4)	287,288 (7.4)	0.91±0.17	3.47 (.001)	5.21 (.002)
	High	128 (14.8)	638,551 (16.4)	0.90±0.14	3.10 (.002)	
	Low	227 (26.2)	991,669 (25.5)	0.89±0.14	1.93 (.055)	
	Very low	446 (51.6)	1,970,301 (50.7)	0.84±0.18		
Economic activity	Yes	256 (29.5)	1,157,951 (29.7)	0.89±0.14	2.26 (.025)	
	No	611 (70.5)	2,740,946 (70.3)	0.85±0.17		
Smoking	Yes	24 (2.8)	125,580 (3.2)	0.86±0.14	0.34 (.734)	
	No	842 (97.2)	3,769,363 (96.8)	0.87±0.17		
Drinking	Yes	314 (36.3)	1,431,448 (36.8)	0.89±0.14	0.19 (.852)	
	No	552 (63.7)	2,463,494 (63.2)	0.86±0.18		
Walking days per week		4.45±2.75			7.91 (<.001)	
*Characteristics of the environment*						
Living alone	Yes	275 (31.7)	1,068,489 (27.4)	0.83±0.19	–3.43 (.001)	
	No	593 (68.3)	2,833,720 (72.6)	0.88±0.15		
*Biological and physiological variables*						
Number of diseases		2.28±1.52			–8.15 (<.001)	
*Symptom status*						
Stress	Very high	38 (4.4)	141,820 (3.6)	0.74±0.26	–3.38 (.001)	7.83 (<.001)
	High	156 (18.0)	713,465 (18.3)	0.83±0.18	–1.81 (.071)	
	Low	444 (51.3)	2,017,528 (51.8)	0.88±0.15	1.21 (.227)	
	Very low	228 (26.3)	1,022,131 (26.3)	0.88±0.16		
*Functional status*						
Activity limitation	Yes	149 (17.2)	555,468 (14.2)	0.72±0.21	–7.54 (<.001)	
	No	719 (82.8)	3,346,742 (85.8)	0.90±0.14		
*General health perceptions*						
Perceived health status	Good	175 (20.2)	796,361 (20.4)	0.94±0.09	11.45 (<.001)	65.39 (<.001)
	Fair	434 (50.0)	2,028,907 (52.0)	0.91±0.11	9.67 (<.001)	
	Poor	259 (29.8)	1,076,942 (27.6)	0.75±0.21		

**Table 2.** Factors associated with different levels of health-related quality of life (N=868)

Variable	Categories	B (*p*)			
		0.1	0.25	0.5	0.75
Characteristics of the individual	Age*	–0.00 (.888)	0.02 (.188)	0.03 (.007)	0.00 (.108)
	Education				
	Elementary or below	–0.05 (.016)	–0.02 (.335)	0.00 (.870)	0.00 (.620)
	Middle school	–0.02 (.349)	0.01 (.666)	0.01 (.457)	0.00 (.051)
	Economic status*				
	Very high	0.06 (.045)	0.05 (.072)	0.01 (.617)	0.00 (.505)
	High	0.01 (.818)	0.02 (.250)	–0.00 (.886)	0.00 (.947)
	Low	0.01 (.685)	0.01 (.539)	0.01 (.423)	0.00 (.116)
	Economic activity*	0.04 (.010)	0.01 (.338)	0.02 (.043)	0.00 (.195)
	Smoking*	–0.00 (.955)	0.06 (.136)	–0.00 (.982)	–0.01 (.008)
	Drinking*	0.01 (.714)	0.01 (.445)	–0.01 (.633)	–0.00 (.407)
	Walking days per week	0.01 (<.001)	0.01 (<.001)	0.01 (.001)	0.01 (<.001)
Characteristics of the environment	Living alone*	–0.03 (.120)	0.01 (.682)	–0.02 (.082)	–0.00 (.105)
Biological and physiological variables	Number of diseases	–0.01 (.021)	–0.02 (<.001)	–0.01 (<.001)	–0.01 (<.001)
Symptom status	Stress*				
	Very high	–0.12 (.002)	–0.06 (.061)	–0.10 (<.001)	–0.04 (<.001)
	High	–0.02 (.494)	–0.01 (.619)	–0.03 (.070)	–0.00 (.785)
	Low	0.00 (.843)	–0.00 (.867)	0.01 (.643)	–0.00 (.281)
Functional status	Activity limitation*	–0.19 (<.001)	–0.12 (<.001)	–0.10 (<.001)	–0.09 (<.001)
General health perceptions	Perceived health status*				
	Good	0.22 (<.001)	0.14 (<.001)	0.10 (<.001)	0.09 (<.001)
	Fair	0.19 (<.001)	0.11 (<.001)	0.08 (<.001)	0.09 (<.001)
Pseudo R-square		.33	.24	.21	.08

* Reference variables were age (≥75), education (high school or above), economic status (very low), economic activity (no), smoking (no), drinking (no), live along (no), stress (very low), activity limitation (no), and perceived health status (poor).
